# Whole Body Vibration Therapy after Ischemia Reduces Brain Damage in Reproductively Senescent Female Rats

**DOI:** 10.3390/ijms19092749

**Published:** 2018-09-13

**Authors:** Ami P. Raval, Marc Schatz, Pallab Bhattacharya, Nathan d’Adesky, Tatjana Rundek, W. Dalton Dietrich, Helen M. Bramlett

**Affiliations:** 1 Cerebral Vascular Disease Research Laboratories, Department of Neurology, Leonard M. Miller School of Medicine, University of Miami, Miami, FL 33136, USA; marc.schatz@med.miami.edu (M.S.); pallab.bhu@gmail.com (P.B.); nathandadesky@gmail.com (N.d.); 2 Department of Neurology, University of Miami School of Medicine, Miami, FL 33136, USA; TRundek@med.miami.edu; 3 Department of Neurological Surgery, Leonard M. Miller School of Medicine, University of Miami, Miami, FL 33136, USA; ddietrich@med.miami.edu; 4 Bruce W. Carter Department of Veterans Affairs Medical Center, Miami, FL 33125, USA

**Keywords:** brain-derived neurotrophic factor, frailty, inflammasome proteins, interleukin-1β, peri-infarct area

## Abstract

A risk of ischemic stroke increases exponentially after menopause. Even a mild-ischemic stroke can result in increased frailty. Frailty is a state of increased vulnerability to adverse outcomes, which subsequently increases risk of cerebrovascular events and severe cognitive decline, particularly after menopause. Several interventions to reduce frailty and subsequent risk of stroke and cognitive decline have been proposed in laboratory animals and patients. One of them is whole body vibration (WBV). WBV improves cerebral function and cognitive ability that deteriorates with increased frailty. The goal of the current study is to test the efficacy of WBV in reducing post-ischemic stroke frailty and brain damage in reproductively senescent female rats. Reproductively senescent Sprague-Dawley female rats were exposed to transient middle cerebral artery occlusion (tMCAO) and were randomly assigned to either WBV or no-WBV groups. Animals placed in the WBV group underwent 30 days of WBV (40 Hz) treatment performed twice daily for 15 min each session, 5 days each week. The motor functions of animals belonging to both groups were tested intermittently and at the end of the treatment period. Brains were then harvested for inflammatory markers and histopathological analysis. The results demonstrate a significant reduction in inflammatory markers and infarct volume with significant increases in brain-derived neurotrophic factor and improvement in functional activity after tMCAO in middle-aged female rats that were treated with WBV as compared to the no-WBV group. Our results may facilitate a faster translation of the WBV intervention for improved outcome after stroke, particularly among frail women.

## 1. Introduction

A woman’s risk of a stroke increases exponentially following the onset of menopause, and even a mild-ischemic episode can result in a woman becoming increasingly frail with age. Frailty is characterized by an increased vulnerability to acute stressors and the reduced capacity of various bodily systems due to age-associated physiological deterioration [1]. Therefore, older women are more likely to experience decreased energy and strength, weight loss, increased susceptibility to disease and physical injury, increased hospitalization, and reduced daily living activities. Our understanding of the link between frailty and cerebrovascular diseases is limited [1]. Thus, understanding the factors that contribute to frailty in women could potentially allow for preventative measures that could decrease or slow down its onset, reduce risk of stroke and provide the basis for new treatment options. 

Exercise is a powerful behavioral intervention that has the potential to improve health outcomes in elderly stroke survivors. Multiple studies using human and animal models have shown that pre-ischemic physical activity reduces stroke impact on functional motor outcomes, edema, and infarct volume. The same studies also attributed these benefits to the mechanism of decreasing inflammation, and increasing brain-derived neurotrophic factor (BDNF) expression [2,3,4,5,6]. In many cases, however, stroke patients are unable to adhere to the physical activity regimen following their ischemic episodes due to a wide range of individual factors such as stroke severity, preexisting and comorbid conditions, motivation, fatigue, and depression. As a result, whole body vibration, a procedure mimicking exercise, has been proposed as an alternative to physical therapy [7]. Whole body vibration (WBV) is a novel rehabilitative exercise that uses low amplitude, low frequency vibration administered through a platform or Power Plate. WBV shows potential as an effective therapeutic approach and has been studied in a variety of clinical settings that include rehabilitation of patients with chronic stroke [8], spinal cord injury [9], lumbar disk disease and lower back pain syndromes [10], Parkinson’s disease [11], elderly with sarcopenia [12,13], chronic obstructive pulmonary disease (COPD) [14], multiple sclerosis [15], obesity, osteoporosis, osteoarthritis and fibromyalgia [16] and children with cerebral palsy [17]. A growing body of evidence in laboratory animals and patients with chronic stroke has shown that WBV reduces or reverses pathological remodeling of bone and such a treatment could also help reduce frailty-related physiological deterioration [18,19,20]. Although WBV has shown to be an effective therapy under many different conditions, its specific application in stroke remains unclear. Several studies of WBV in stroke patients [21,22], of which none were specifically screened for frailty or pre-frailty, have produced inconclusive results [23]. Also, WBV has not yet been systematically studied specifically in women who are often more critically affected by stroke than men. Therefore, the goal of our current study is to investigate the effect of WBV on ischemic outcome in the reproductively senescent (RS) female rat model. Our selection of using a RS female rat model in this study is also adhering to Stroke Therapy Academic and Industry Roundtable (STAIR) guidelines that recommend more relevant animal models to better correlate with the aged population. Based on the currently available literature, we hypothesize that the benefit observed from WBV will be similar in mechanism to the one followed by physical therapy—reducing inflammation and increasing BDNF—resulting in reduced post-ischemic injury, improved activity and neurobehavior in reproductively senescent female rats. These results would serve as preliminary translational data for adoption in a clinical trial of pre-frail and frail women after stroke. 

## 2. Results

### 2.1. Post-Ischemic WBV Reduced Infarct Volume in Middle-Aged Female Rats

**Figure 1:**
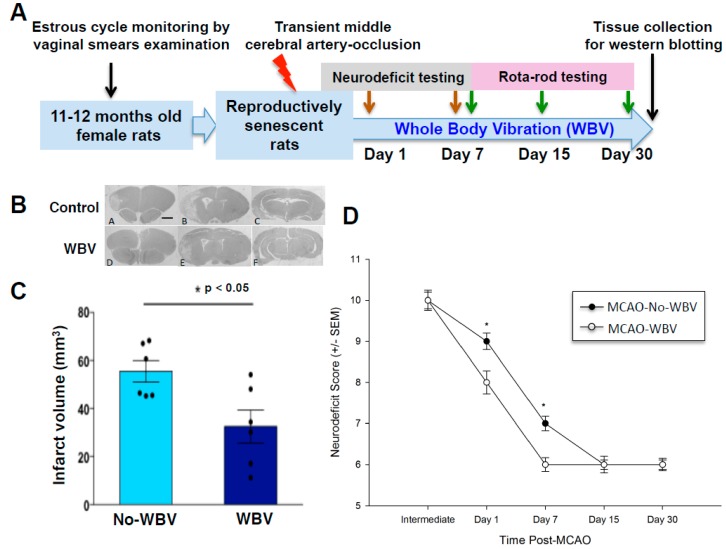
(**A**) Experimental design. (**B**) Representative histological images of the brain (Bregma levels 1.2, −3.8, −5, 10X). (**C**) Geometric mean infarct volumes are compared between whole body vibration WBV and no-WBV groups. Post-ischemic WBV treatment shows reduced infarct volume as compared to the no-WBV group (* *p* < 0.05 as compared to no-WBV using student *t*-test). (**D**) Neurological deficit (ND) assessment scores were significantly improved in the WBV treated group as compared to no-WBV (* *p* < 0.05 as compared to no-WBV using Student Newman-Keuls).

Our first hypothesis was that post-ischemic WBV reduced infarct volume. Rats exposed to transient middle cerebral artery occlusion (tMCAO) were treated with WBV or no-WBV and a month later, brain tissue was collected for histopathological assessment (Figure 1A). The results demonstrate a significant reduction in infarct volume in a mild stroke model following WBV treatment as compared to no-WBV rats (Figure 1B,C). We observed a 41% reduction in infarct volume of WBV treated rats as compared to no-WBV. Histological analysis of WBV or no-WBV-treated rat brains that underwent sham surgery did not show any infarct. In parallel, we also monitored neurological deficit of rats that were exposed to WBV/no-WBV treatment after tMCAO (Figure 1D). Results demonstrated a significant improvement in the neurological score following WBV as compared to no-WBV rats.

### 2.2. Post-Ischemic WBV Improved Neuro-Deficit Score and Motor Function in Middle-Aged Female Rats

**Figure 2:**
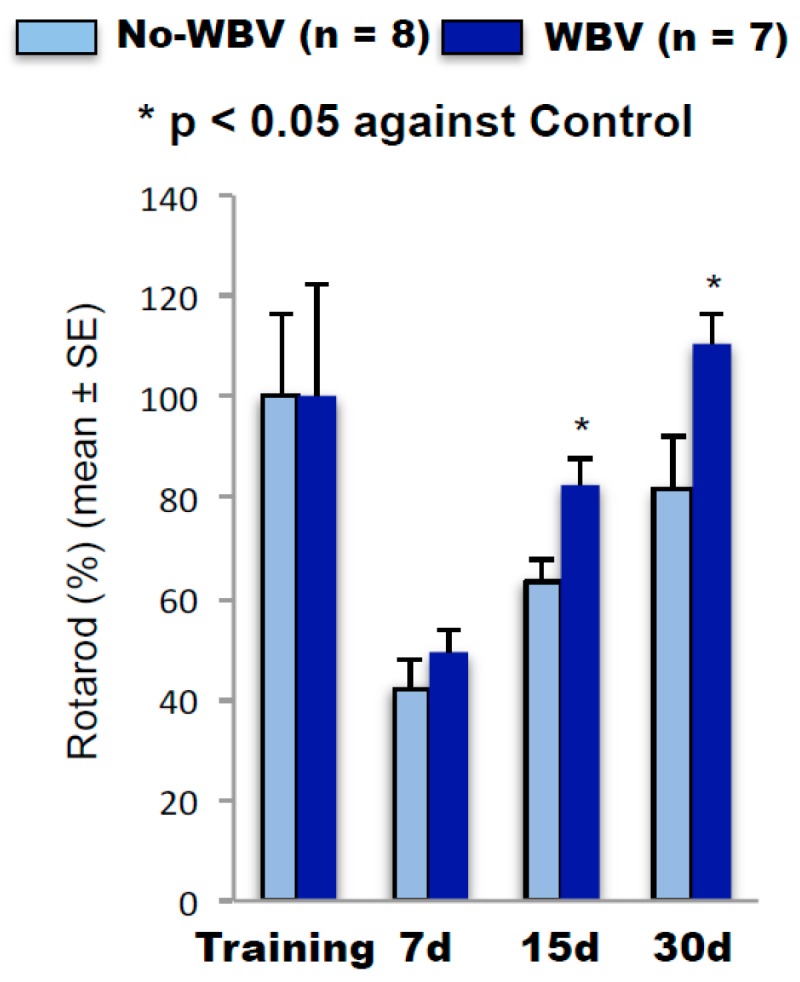
Post-ischemic WBV improves motor coordination (* *p* < 0.05 as compared to no-WBV using student *t*-test).

Secondly, we tested the hypothesis that post-tMCAO WBV treatment improves neurodeficit and motor coordination along with an observed reduction in ischemic damage. The neurodeficit score in each group was more than 9 at baseline when tested at 1 h after tMCAO. Over the period of 7 days, the neurodeficit score was reduced significantly in rats that were treated with WBV (*p* < 0.05) after tMCAO as compared with corresponding no-WBV-treated groups. The rotarod test scores from rats receiving WBV treatment as compared to no-WBV group were significantly higher on day 30 (*p* < 0.05) at 10, 30, and 40 rotations per minute (rpm) speed. These results demonstrate a significant improvement in functional activity after tMCAO in animals that were treated with WBV as compared to the no-WBV group (Figure 2).

### 2.3. Post-Ischemic WBV Decreased Inflammasome Activation in the Brain of Middle-Aged Female Rats

**Figure 3:**
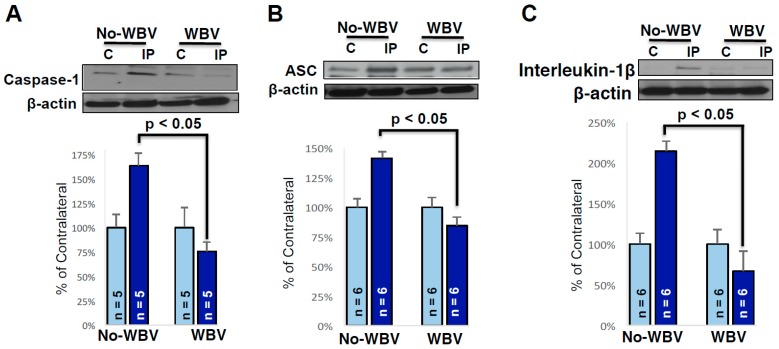
Representative immunoblots showing the protein levels of caspase 1 (**A**-Top), ASC (**B**-Top), and IL-1β (**C**-Top), in the contra-lateral and ipsilateral peri-infarct region of the brain, respectively. Post-ischemic WBV decreases inflammasome proteins caspase 1 (**A**-Bottom), ASC (**B**-Bottom), and IL-1β (**C**-Bottom), in the contra-lateral and ipsilateral peri-infarct region of the brain, respectively (* *p* < 0.05 as compared to no-WBV using student *t*-test).

Western blot results demonstrated a two-fold decrease in the inflammasome proteins caspase-1, caspase recruitment domain (ASC), and interleukin-1β in the peri-infarct area of WBV treated rats. Since the peri-infarct area is salvageable tissue after stroke, for this study, we focused on investigating alterations in inflammasome proteins in the peri-infarct area of WBV treated versus the no-WBV rats (Figure 3). Post-ischemic WBV decreased protein levels of caspase-1, ASC and IL-1β by 88% (*p* < 0.05), 57% (*p* < 0.05) and 148% (*p* < 0.05) in peri-infarct area as compared to no-WBV-treated group.

### 2.4. Post-Ischemic WBV Increased Brain-Derived Growth Factor (BDNF) and Trk-B Protein Levels in the Peri-Infarct Area

**Figure 4:**
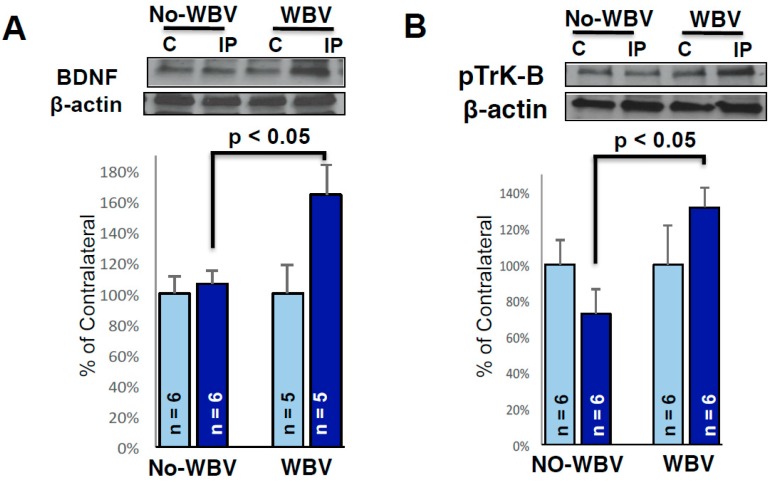
Representative immunoblots showing the protein levels of BDNF and phosphorylated Trk-B in the peri-infarct area. β-actin (cytoskeletal), was used as a loading control. Densitometric analysis of scanned Western blots and expressed as percent of contralateral, showed baseline expression of BDNF (**A**) and phosphorylated Trk-B (**B**) proteins. Note the WBV treatment significantly increased BDNF and phosphorylated Trk-B in the peri-infarct area as compared to no-WBV (* *p* < 0.05 as compared to no-WBV using student *t*-test).

Studies from various laboratories demonstrate that growth factors play an important role in preserving brain function after ischemia. Therefore, we tested whether WBV treatment after tMCAO increases BDNF release and tyrosine kinase receptor subtype B (Trk-B) signaling in the female brain. We observed significant increases in levels of BDNF and pTrK-B in the peri-infarct region of WBV treated group as compared to the no-WBV (Figure 4). Post-ischemic WBV increased protein levels of BDNF and pTrk-B by 58% (*p* < 0.05) and 59% (*p* < 0.05) in peri-infarct area as compared to no-WBV-treated group.

## 3. Discussion

The current study demonstrates that the post-stroke WBV intervention reduces brain injury in reproductively senescent female rats. Our study also demonstrated that the post-stroke WBV intervention significantly improved neurological and motor capabilities in female rats. The mechanism by which the WBV intervention improved outcomes after stroke is likely multi-factorial, similar to that of exercise. The benefits of post-stroke exercise go beyond reduced infarct volume and have shown to improve motor and cognitive functions. Studies in recent years demonstrate that physical exercise has a profound effect on the normal functioning of the immune system [24,25,26]. Moderate intensity exercise was shown to be beneficial for immunity, which could be the result of reduced inflammation, thymic mass maintenance, changes in immune cells’ compositions, increased immunosurveillance, and/or amelioration of psychological stress [24,25,26]. It is well known that exercise is an important intervention that can improve immunity and health outcomes in elderly stroke survivors. However, after stroke, patients are unable to exercise or less likely to adhere to the physical activity regimen following their ischemic episodes. A wide range of individual factors may affect stroke patient participation in physical therapy including stroke severity, preexisting and comorbid conditions, motivation, fatigue, and depression. Therefore, the current approach to reduce post-stroke inflammation and frailty using WBV has important translational value.

The current study demonstrated that post-stroke WBV reduces pro-inflammatory cytokine IL-1β and inflammasome proteins in the brain in middle-aged female rats. The importance of inflammasome as a key component of the innate immune response in brain injury has been recently emphasized and targeted for therapeutic interventions [27,28,29,30]. Specifically, the inflammasome was shown to activate caspase-1 and initiate the processing of the inflammatory cytokines IL-1β and IL-18 [31]. In models of brain ischemia, evidence for inflammasome activation has been reported with elevations in inflammatory proteins such as ASC, and caspase-1. Our previously published studies demonstrated elevations in inflammasome proteins in the hippocampus of aged rats [32,33]. Consistent with our findings, others have demonstrated increased pro-inflammatory cytokine levels in middle-aged female rats [34]. It is now well documented that the depletion of estrogens at menopause/reproductive senescence elevates pro-inflammatory cytokines, which may increase the chances of inflammatory diseases in the body, including the brain. This decline in estrogen is also associated with a loss of muscle mass, bone, and strength that represent the core of the frailty syndrome [35,36]. Our use of reproductively senescent female rats closely mimics the age group of peri-menopausal women and the population that is likely to suffer frailty following stroke. Therefore, showing benefits of post-stroke WBV in reducing inflammation in the brain is of a translational value.

Since post-ischemic inflammation eventually subsides while injured tissue undergoes structural and functional reconstruction, this process may further require the release/presence of variety of growth factors such as BDNF [37]. In our current study, we observed significant increases in levels of BDNF and pTrK-B in the peri-infarct region after WBV. BDNF, a member of the neurotrophic factor family, is one of the most powerful neuroprotective agents [38,39,40]. BDNF expression is regulated in an activity-dependent manner by physiological stimuli, and its biological effects are mediated through the high-affinity receptor, tyrosine kinase receptor subtype B (Trk-B) [41]. Since BDNF expression is augmented in neurons by various stressors (e.g., ischemia, epilepsy, hypoglycemia, and trauma [42]), chronic exposure to BDNF confers neuroprotection. In addition to pro-survival mechanism(s), BDNF also modulates synaptic plasticity and neurogenesis [43,44,45,46]. A direct application of BDNF is neuroprotective in focal and global cerebral ischemia models [47,48]. Importantly, continuous intraventricular administration of BDNF was required for mitigating ischemic brain damage in the aforementioned in vivo studies. Despite BDNF’s neuroprotective ability against ischemic damage, treating patients with BDNF remains challenging because BDNF is unable to cross the blood-brain barrier [49,50]. Due to the difficulty of administering BDNF directly to the brain, a model in which BDNF is increased intrinsically has been proposed. Several studies have shown a strong correlation between increased levels of circulating BDNF and exercises, yet no studies have shown an increase in BDNF levels with WBV. One study has shown that exercise in mice is effective at preventing a decrease in BDNF levels in the CA1 and dentate gyrus that would otherwise be caused by exposure to Arsenic [51]. It is proposed that training to volatile fatigue is the optimal way to increase circulating BDNF levels in elderly participants [52]. Intravenous BDNF delivery enhances post-stroke sensorimotor recovery and stimulates neurogenesis [53]. It has also been demonstrated that BDNF up-regulation following exercise is associated with a robust activation of survival pathways that enhance adult neurogenesis in experimental animals [54,55]. Currently, it is unknown whether WBV leads to increases in hippocampal BDNF and whether this response promotes neurogenesis associated with improved cognitive outcome after stroke, but we suspect that this may be the missing link between WBV and exercise.

The caveats of the current study are that (1) it lacks a mechanistic approach to prove the role of either inflammation or BDNF in WBV-mediated ischemic protection, and (2) the effects of post-stroke WBV are only tested on RS female rats. Therefore, the observed improvement in motor function and reduced infarct volume could not be generalizable to both rat sexes.

In conclusion, the results of our study demonstrated that the post-ischemic WBV intervention reduces brain injury and frailty in reproductively senescent female rats, suggesting WBV may be a potential therapy to reduce post-ischemic frailty and improve functional and cognitive outcomes in women after stroke. Our use of reproductively senescent female rats closely mimics the age group of peri-menopausal women and is clinically relevant as it is estimated that 7 million American adults are living with a stroke and the majority of them are post-menopausal women. This is particularly important because we now know that stroke disproportionately kills more women than men. Although women are naturally protected against stroke in their pre-menopausal life, a woman’s risk of stroke increases exponentially after menopause. The decline in ovarian hormones, especially estrogen, at menopause is associated with loss of muscle mass, bone and strength that represents the core of the frailty syndrome [35,36]. Whole body vibration as a simple and an inexpensive intervention that can be administered at homes has a great potential to aid in prevention and treatment of post-stroke frailty. Future pre-clinical studies investigating the specific mechanism of post-stroke frailty and efficacy of WBV in improving post-stroke frailty and other stroke outcomes can lead to its clinical translation.

## 4. Materials and Methods

All animal procedures were carried out in accordance with the Guide for the Care and Use of Laboratory Animals published by the U.S. National Institutes of Health and were approved (protocol # 17-034; 03-08-2017) by the Animal Care and Use Committee of the University of Miami, University of Miami, Florida, USA. Retired breeder (9–12 months) Sprague-Dawley female rats (280–350 g) were purchased, and their estrous cycles were checked for 14–20 days before experimentation by daily vaginal smears [56]. Rats that persisted in a single stage for 7 days were considered acyclic. The acyclic rats and rats that remained in constant diestrous were considered reproductively senescent (RS) and were used in the study [57].

Reproductively senescent rats were randomly exposed to 60 min of transient middle cerebral artery occlusion (tMCAO) or sham surgery. Transient MCAO was adapted from previous publications [58,59]. tMCAO was achieved by intraluminal suture. A 30-mm-long 3-0 nylon monofilament suture coated with silicone (Doccol) and was placed 19–20 mm into the internal carotid artery to occlude the ostium of the MCA. The suture was placed in the MCA for 60 min and the drop in cerebral blood pressure was confirmed using laser Doppler (LDF, Perimed Inc., Ardmore, PA, USA). For sham surgical procedure, rats were exposed to anesthesia for a period similar to that of the tMCAO group. Physiological parameters including, pCO_2_, pO_2_, and pH were maintained within normal limits through the surgery or sham-surgery. Mean arterial blood pressure (MABP) was continuously monitored and head and body temperatures were maintained at 37 °C.

One day after the tMCAO, animals were randomly assigned to (1) a WBV intervention group or to (2) a no-WBV group. Animals randomized to the WBV group underwent 30 days of treatment performed twice daily for 15 min each session, 5 days each week. The vibration device was programmed in order to achieve a frequency of vibration within a range of about 40 Hz (0.3 g) similar to those used in clinical studies [9,60,61]. The duration and frequency of sessions were selected based on our recent publication [18], where we demonstrated an ability of WBV to improve selected biomarkers of bone turnover and gene expression and to reduce osteoclastogenesis after spinal cord injury. The no-WBV animals post tMCAO were also placed on the platform with no activation. To provide WBV intervention, animals were placed in a plexiglass box that contained four chambers. One rat was placed into each chamber in a random order from one session to the next to avoid any bias due to chamber placement. The vibration parameters were measured in each chamber and differences in these parameters between the chambers were negligible.

Rats exposed to WBV or no-WBV treatment after tMCAO were allowed to survive for a month for histopathological assessment. At one month, rats were anesthetized and perfused via the ascending aorta with FAM (a mixture of 40% formaldehyde, glacial acetic acid, and methanol, 1:1:8 by volume) for 20 min after first being perfused for 2 min with saline. The rat heads were immersed in FAM for 1 day before the brains were removed. The brains were kept in FAM at 4 °C for at least 1 additional day, and then coronal brain blocks were fixed in paraffin. All brains were cut into 10-μm thick sections from 5.5 mm to −7.5 mm from bregma at 9 standard levels to span the entire infarcted area. Sections of the 9 levels were stained with hematoxylin and eosin to visualize the infarcted areas and to calculate infarct volumes. The electronic images of the tissue sections were obtained using a CCD camera and infarct volume was quantified using an MCID image analysis system [62].

### 4.1. Neurodeficit Sscoring and Motor Deficit Test

A standardized neurobehavioral test battery was conducted as described previously [62]. This test consists of quantifications of postural reflex, sensorimotor integration and proprioception. Total neurodeficit score ranged from a score of 0, indicating normal results, to a maximal possible score of 12, indicating a severe deficit.

To further test motor function, we performed the rotarod test as described in our previous publication [63]. In this test, the rats were placed on the rotarod cylinder, and the time that animals remained on the rotarod was measured. The speed was slowly increased from 10 to 40 rpm over 5 min. The trial ended if a rat fell off of the device or spun around for 2 consecutive revolutions without the rat attempting to walk. The rats were trained for 3 consecutive days before undergoing the MCAO procedure. The average duration (in seconds) on the machine was recorded from 3 different rotarod measurements 1 day prior to surgery. Motor function data are presented as percentage of mean duration (3 trials) on the rotarod compared to the internal baseline control (before surgery). The rats were tested at 1, 15, and 30 days after MCAO.

### 4.2. Immunoblot Analysis

Brain tissue was harvested 30 days after WBV or no-WBV. We isolated the peri-infarct and corresponding contralateral region of the brain for the analysis of the WBV or no-WBV groups and tissues were stored at −80 °C. At the time of immunoblotting, tissues were homogenized; protein content was analyzed and proteins were separated by 12% SDS-PAGE as described [56]. Proteins were transferred to Immobilon-P (Millipore, Burlington, MA, USA) membrane and incubated with primary antibodies against caspase-1 (mouse monoclonal; 1:1000; Novus Biologicals, Littleton, CO, USA), ASC (mouse monoclonal; 1:1000; Santa Cruz Biotechnology, Santa Cruz, CA, USA), IL-1β (1:1000, Cell Signaling, Danvers, MA, USA), BDNF (rabbit polyclonal; 1:500; Santa Cruz Biotechnology, Santa Cruz, CA, USA) and Trk-B (rabbit polyclonal; 1:500; Santa Cruz Biotechnology, Santa Cruz, CA, USA). All data were normalized to β-actin (monoclonal; 1:1000; Sigma, St. Louis, MO, USA). Immunoblot images were digitized and subjected to densitometric analysis [56].

### 4.3. Statistical Analysis

The data are shown as the mean value ± SEM or median ± SEM, and the results from the densitometric analysis were analyzed by a two-tailed Student’s *t*-test. The neurodeficit score was analyzed with a two-way repeated measures ANOVA followed by Student Newman Keuls test. A *p* < 0.05 was considered statistically significant.
